# Impact of age on premenstrual syndrome prevalence and severity: A population‐based survey in Brazil

**DOI:** 10.1002/ijgo.15895

**Published:** 2024-09-25

**Authors:** Adriana Orcesi Pedro, Juliana Dineia Perez Brandão, Samantha Belamarques de Oliveira Silva, Maura Gonzaga Lapa, Vivienne Carduz Castilho

**Affiliations:** ^1^ Department of Gynecology and Obstetrics, Universidade Estadual de Campinas (UNICAMP) Campinas Brazil; ^2^ Libbs Farmacêutica Ltda Scientific Medical Division São Paulo Brazil

**Keywords:** Brazilian women, physical symptoms, premenstrual syndrome, psycho‐emotional symptoms, survey

## Abstract

**Objective:**

To evaluate the relationship between premenstrual disorders (PMD) and age, we analyzed the prevalence and severity of psycho‐emotional and physical symptoms in a representative sample of Brazilian women.

**Methods:**

This observational and retrospective study analyzed data from Brazilian women aged 20–49 years from five regions of the country who reported premenstrual symptoms. Participants completed a premenstrual symptom screening questionnaire and self‐reported the presence and severity of their symptoms. Among 23 104 women reporting does premenstrual symptoms, 38.91% (*n* = 8990) experienced PMD caused functional impairment. Finally, 5121 participants agreed to complete the adapted version of the screening questionnaire.

**Results:**

The age group distribution was 20–29 (46.7%), 30–39 (38.3%), and 40–49 years (15%). The most prevalent and severe physical symptom was acne/oily skin in participants aged 20–29 years and headache in women aged 30–49 years. Regarding psycho‐emotional symptoms, the most prevalent was anxiety/tension in women aged 20–29 years and 40–49 years and irritability/anger in those aged 30–39 years. Irritability/anger was the most severe symptom in all groups.

**Conclusion:**

PMD significantly impacts the quality of life of Brazilian women with varying intensity. Physical symptoms associated with PMD vary with age, while psycho‐emotional symptoms, particularly irritability/anger and anxiety, were intense in Brazilian women of reproductive age. These findings inform early diagnosis and individualized treatment approaches for PMD, addressing the needs of women.

## Synopsis

The aim of the study was to assess the prevalence and severity of premenstrual disorders in Brazilian women aged 20–49, elucidating age‐related symptom variations and their impact on quality of life.

## 1 INTRODUCTION

Premenstrual syndrome (PMS), now termed premenstrual disorder (PMD), encompasses psycho‐emotional and/or physical symptoms during the luteal phase, significantly impacting women's quality of life and daily functioning. The most severe form of PMD is premenstrual dysphoric disorder (PMDD), characterized by emotional and affective symptoms distinct from other psychiatric conditions.^1^, ^2^ Recently included in the Diagnostic and Statistical Manual of Mental Disorders and in the World Health Organization's International Classification of Diseases 11th revision,^3^ PMDD requires at least one mood symptom, such as marked affective lability, irritability, depressed mood, anxiety, or tension, alongside a group of other symptoms, including loss of interest, subjective difficulty in concentrating, fatigue, marked appetite changes with overeating or food cravings, insomnia or hypersomnia, feeling emotionally overwhelmed, and various physical symptoms.^1^, ^2^, ^3^, ^4^, ^5^, ^6^

The pathophysiology of PMDs remains unclear, with symptoms cyclically varying in intensity and duration.^7^ Diagnosis hinges on symptoms' timing and impact on daily activities during the luteal phase, rather than their specific type. PMDs affect 80%–90% of women, including mild cases, and are associated with sociodemographic, reproductive, and behavioral factors. Global studies aim to comprehend PMD's widespread impact on daily life.^8^, ^9^

A study of 302 women aged 18–45 identified negative Rhesus blood type, age at menarche, caffeine consumption, and self‐reported depression as significant risk factors for PMD and PMDD.^10^ Global meta‐analysis estimated a PMD prevalence of about 47.8%, but variations in study methodologies and limited sample sizes complicate comparisons.^1^

The relationship between age and PMD symptoms remains unclear due to conflicting findings among studies.^11^ Due to contradictory findings and varied study methods in existing research, larger population‐based studies are necessary to clarify the association. A previous meta‐analysis reported that larger sample sizes typically reduce the incidence of PMD.^1^

Limited research on PMD in Brazilian and Latin American populations, mainly address sociodemographic and socioeconomic factors. Therefore, our cross‐sectional study aimed to explore PMD prevalence and symptom severity among Brazilian women of varying ages.

## 2 MATERIALS AND METHODS

### 2.1 Study design

The study protocol, approved by the Research Ethics Committee (registration no. 33794520.1.0000.8098), followed ethical guidelines from Resolution 466/2012, Operational Standard 001/2013, and related resolutions.

This observational, retrospective study utilized anonymized data from the Market Research Programs (MRP) database, ensuring confidentiality and security.

### 2.2 Data collection

Data were collected via self‐reported electronic questionnaires administered from February 2019 to March 2020 to women aged 20–49 years. The study encompassed 303 voluntary private healthcare clinics across 22 Brazilian states and the federal district, covering 168 cities. Participants provided informed consent and completed an adapted version of the premenstrual symptoms screening tool (PSST), validated in Brazil. The PSST, which employs a retrospective, self‐administered format utilizes a four‐point Likert scale to evaluate the severity of psycho‐emotional (irritability, anxiety and tension, decreased interest in routine activities, depression and sadness, overeating, concentration difficulties, emotional instability) and physical symptoms (headache, acne and oily skin, edema, weight gain, breast tenderness, exacerbation of immunoallergic conditions) based on severity (0 = none; 1 = mild; 2 = moderate; 3 = severe).

Participants meeting the diagnostic criteria for PMD included the presence of specific symptoms during the 15 days prior to menstruation over three consecutive menstrual cycles, according to established guidelines from the American College of Obstetricians and Gynecologists (AGOC) and the Royal College of Obstetricians and Gynecologists (RCOG). Participants who answered, “Bothers a lot” to the question “How much do the mentioned symptoms interfere with your daily life?” and reported at least one or more psycho‐emotional or somatic symptoms were included.

### 2.3 Variables and diagnostic criteria

The independent variables included age, city and state of residence, intensity of self‐reported physical and psycho‐emotional symptoms using the PSST, (categorized as absent, mild, moderate, and severe) and contraceptive methods.

The dependent variable was the presence of diagnostic criteria for PMD, defined by the International Society for Premenstrual Syndrome/Disorders, ACOG and RCOG. The criteria required: (1) one or more affective or somatic symptoms during the 15 days before menstruation in each of the three previous menstrual cycles; (2) affective symptoms: depression, anger, irritability, anxiety, binge eating, decreased interest, and difficulty concentrating; (3) somatic symptoms: mastalgia, abdominal distension, headache, edema of the extremities, acne/skin oiliness, and immunoallergic reaction and (4) significant impairment in performance, daily activities, interpersonal relationships, social, or economic aspects.

### 2.4 Sample selection methodology

**FIGURE 1:**
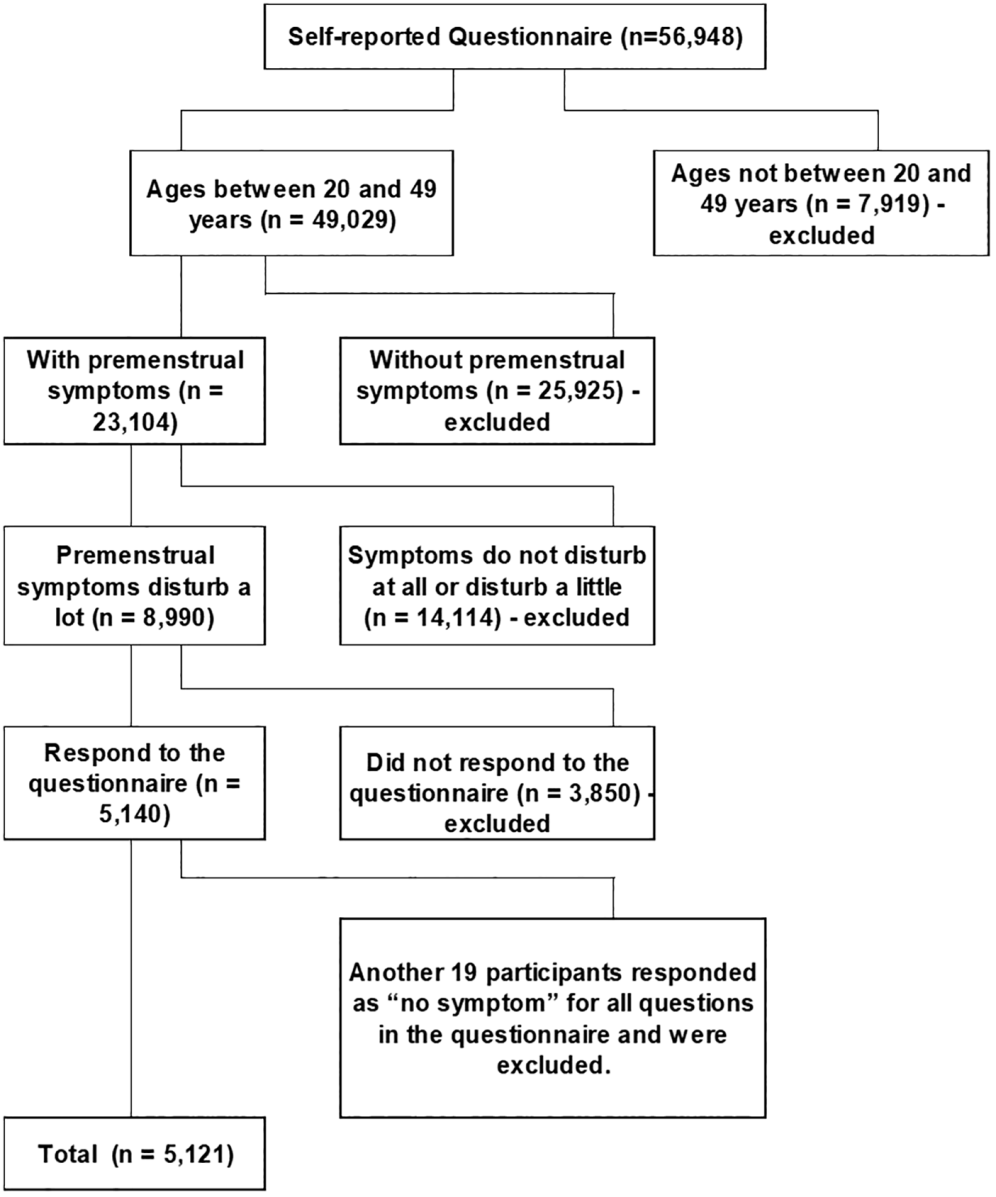
Flow chart for participants' self‐reported questionnaire and final sample size.

The questionnaire was administered to 56 948 Brazilian women. Of these, 49 029 were aged 20–49 years and met the inclusion criteria. Among these, 23 104 reported PMS symptoms, but only 8990 experience functional impairment, qualifying for PMD. Of these, 5140 women complete the questionnaire, with 19 excluded for reporting no PMD symptoms, resulting in a final sample of 5121 women (Figure 1).

Participants accessed the survey through the clinic's wireless network on an electronic device. Those who agreed to participate voluntarily were directed to a consent form and filled out an adapted PSST questionnaire. The PSST was offered only to women reporting PMD symptoms with functional impairment in their daily routine. The data were anonymized and stored in the cloud.

### 2.5 Sample calculation

For this descriptive study with categorical qualitative variable, the sample size was calculated using an estimation formula, with the estimate for PMD obtained from the literature.^12^ The significance level (alpha) was set at 95% confidence intervals (CI), and the sampling error was set at 3% (d = 0.03). Based on these parameters, a minimum sample size of *n* = 1022 was required. The analysis was conducted using SAS version 9.4 (SAS Institute Inc, 2002–2012, Cary, NC, USA) and Excel.

### 2.6 Statistical analysis

Frequency tables with absolute (n) and percentage (%) frequencies were generated for categorical variables. Pearson's chi‐square test compared age groups, with significant difference (*P* < 0.05). Significant differences, two‐by‐two (multiple) comparisons using Bonferroni multiplicity correction (*P* < 0.017) were used to adjust for three age groups. Poisson regression was used to evaluate the percentage of women reporting moderate or severe symptoms by age group, utilizing SAS version 9.4 and Excel for analyses.

## 3 RESULTS

Among the 8990 women meeting the PMD criteria, 5121 agreed to complete the PSST questionnaire. The majority, 2391 women (46.7%), were aged 20–29 years followed by 1961 (38.3%) in the 30–39 age group, and 769 (15%) in the 40–49 age group. The participants were distributed across Brazil as follows: North (198, 3.9%), Midwest (258, 5.0%), South (501, 9.8%), Northeast (666, 13.0%), and Southeast (3498, 68.3%). There was no significant difference in age distribution between respondents and non‐respondents across regions (*P* > 0.050).

### 3.1 Physical symptoms

The most prevalent physical symptoms among participants aged 20–29 years were acne/oily skin (2113 cases, 88.3%), edema (1974 cases, 82.5%), and headache (1939 cases, 81.0%). Acne/oily skin was also the most severe symptom reported by this age group (Table 1).

**TABLE 1:** Prevalence of physical symptoms by age group.

Prevalent physical symptoms	20–29 years (*n* = 2394)	30–39 years (*n* = 1959)	40–49 years (*n* = 768)	Total (*n* = 5121)	*P*‐value
Headache					
Total^a^	81.0%^b^	89.5%	90.5%	85.7%	*P* < 0.001
Severe	33.5%^b^	46.6%	48.2%	41.1%	*P* < 0.001
Acne/oily skin					
Total^a^	88.3%^b^	85.2%^b^	78.1%^b^	85.6%	*P* < 0.001
Severe	38.1%^b^	28.3%^b^	20.7%^b^	32.0%	*P* < 0.001
Weight gain					
Total^a^	79.3%^b^	88.9%	89.5%	84.5%	*P* < 0.001
Severe	30.6%^b^	35.4%	36.7%	33.5%	*P* < 0.001
Edema					
Total^a^	82.5%	84.5%	84.8%	83.6%	*P* = 0.117
Severe	23.2%	26.4%	26.3%	24.9%	*P* = 0.054
Breast tenderness					
Total^a^	73.8%^b^	87.1%	87.4%	80.9%	*P* < 0.001
Severe	18.5%^b^	22.1%	23.8%	20.9%	*P* = 0.004
Exacerbation of imunologic conditions					
Total^a^	75.4%^b^	79.4%^b^	77.3%	77.2%	*P* = 0.011
Severe	15.6%	14.1%	15.3%	15.0%	*P* = 0.448

^a^ Total = prevalence of any intensity/prevalence of severe intensity.

^b^ Statistically significant differences between age groups.

For participants aged 30–39 years, the most prevalent physical symptom was headache (1754 cases, 89.5%), followed by weight gain (1742 cases, 88.9%) and breast tenderness (1706 cases, 87.1%). Of these, the most severe symptom was headache affecting 818 women (46.6%) (Table 1).

Among participants aged 40–49 years, the most prevalent symptoms were headache (reported by 695 participants, 90.5%), weight gain (reported by 687 participants, 89.5%), and breast tenderness (reported by 671 participants, 87.4%). Of the symptoms observed, the most severe was headache reported by 335 participants (48.2%) (Table 1).

Overall, the prevalence of physical symptoms generally increased with age, except for acne/oily skin.

### 3.2 Psycho‐emotional symptoms

In participants aged 20–29 years, the most prevalent psycho‐emotional symptoms were anxiety/tension (2355, 98.4%), irritability/anger (2350, 98.2%), and depression/sadness (2251, 94.0%). Among participants aged 30–39 years, irritability/anger, anxiety/tension, and decreased interest in routine activities were the most prevalent psycho‐emotional symptoms, occurring in 1934 (98.7%), 1929 (98.5%), and 1872 (95.6%) participants, respectively.

Of participants in the 40–49 age group, anxiety/tension 761 (99.1.5%), irritability/anger (755, 98.3%) and depression/sadness (740, 96.4%) were prevalent. Overall, psycho‐emotional symptoms were highly prevalent across all age groups, affecting at least 90.8% of participants. Irritability/anger were the most severe symptoms reported in all age groups, with rates 1253 (64.8%), 1934 (98.7%) and 495 (65.6%) observed in the 20–29, 30–39 and 40–49 age groups, respectively (Table 2).

**TABLE 2:** Prevalence of psycho‐emotional symptoms by age group.

Prevalent physico‐emotional symptoms	20–29 years (*n* = 2394)	30–39 years (*n* = 1959)	40–49 years (*n* = 768)	Total (*n* = 5121)	*P*‐value
Irritability/anger					
Total^a^	98.2%	98.7%	98.3%	98.4%	*P* = 0.332
Severe	58.6%^b^	64.8%	65.6%	62.0%	*P* < 0.001
Anxiety/tension					
Total^a^	98.4%	98.5%	99.1%	98.5%	*P* = 0.351
Severe	52.7%	55.0%	62.3%^b^	55.0%	*P* < 0.001
Depression/sadness					
Total^a^	94.0%^b^	93.4%	96.4%^b^	94.5%	*P* = 0.044
Severe	43.8%	45.4%	43.0%	44.3%	*P* = 0.447
Decrease interest in routine activities					
Total^a^	93.1%^b^	95.6%^b^	95.3%	94.4%	*P* = 0.001
Severe	39.7%	36.3%	36.2%	37.9%	*P* = 0.053
Binge eating					
Total^a^	92.1%	93.3%^b^	90.1%^b^	92.2%	*P* = 0.027
Severe	50.1%	48.5%	42.2%^b^	48.3%	*P* = 0.001
Emotional instability					
Total^a^	89.3%^b^	92.1%	93.0%	90.2%	*P* < 0.001
Severe	28.2%^b^	30.2%	36.0%	30.9%	*P* < 0.001
Difficulty concentrating					
Total^a^	90.3%	90.7%	92.8%	90.8%	*P* = 0.102
Severe	22.5%	23.9%	22.9%	23.1%	*P* = 0.599

^a^ Total = prevalence of any intensity/prevalence of severe intensity.

^b^ Statistically significant differences between age groups.

### 3.3 Comparison of severe symptoms between age groups

Participants aged 30 years and older reported a greater prevalence of somatic and psychological symptoms in comparison to the younger women. Specifically, women aged 20–29 years demonstrated significantly lower severity of irritability/anger symptoms (1377, 58.6%), emotional instability (603, 28.2%), headache (649, 33.5%), weight gain (581, 30.6%), and breast tenderness (327, 18.5%) compared to other age groups. However, the prevalence of acne/oily skin was significantly higher in this group (806, 38.1%) was more severe in this group.

Among women aged 30–39 years, acne/oily skin (472, 28.3%) was the only symptom significantly different from the younger group, showing lower severity. In the 40–49 year age group, acne/oily skin (124, 20.7%) and binge eating (292, 42.2%) were less severe, while anxiety/tension (474, 62.3%) was more severe compared to younger age groups.

Significant variations in symptom severity were evident across different age groups. Depression/sadness, decreased interest in routine activities, edema, difficulty concentrating, and exacerbation of immunoallergic conditions showed notable differences in severity. Interestingly, while acne/oily skin decreased significantly with age, the severity of edema and exacerbation of immunoallergic conditions remained consistent across age groups. Overall, irritability/anger, anxiety, and tension consistently emerged as the most severe symptoms across all age cohorts studied.

**FIGURE 2:**
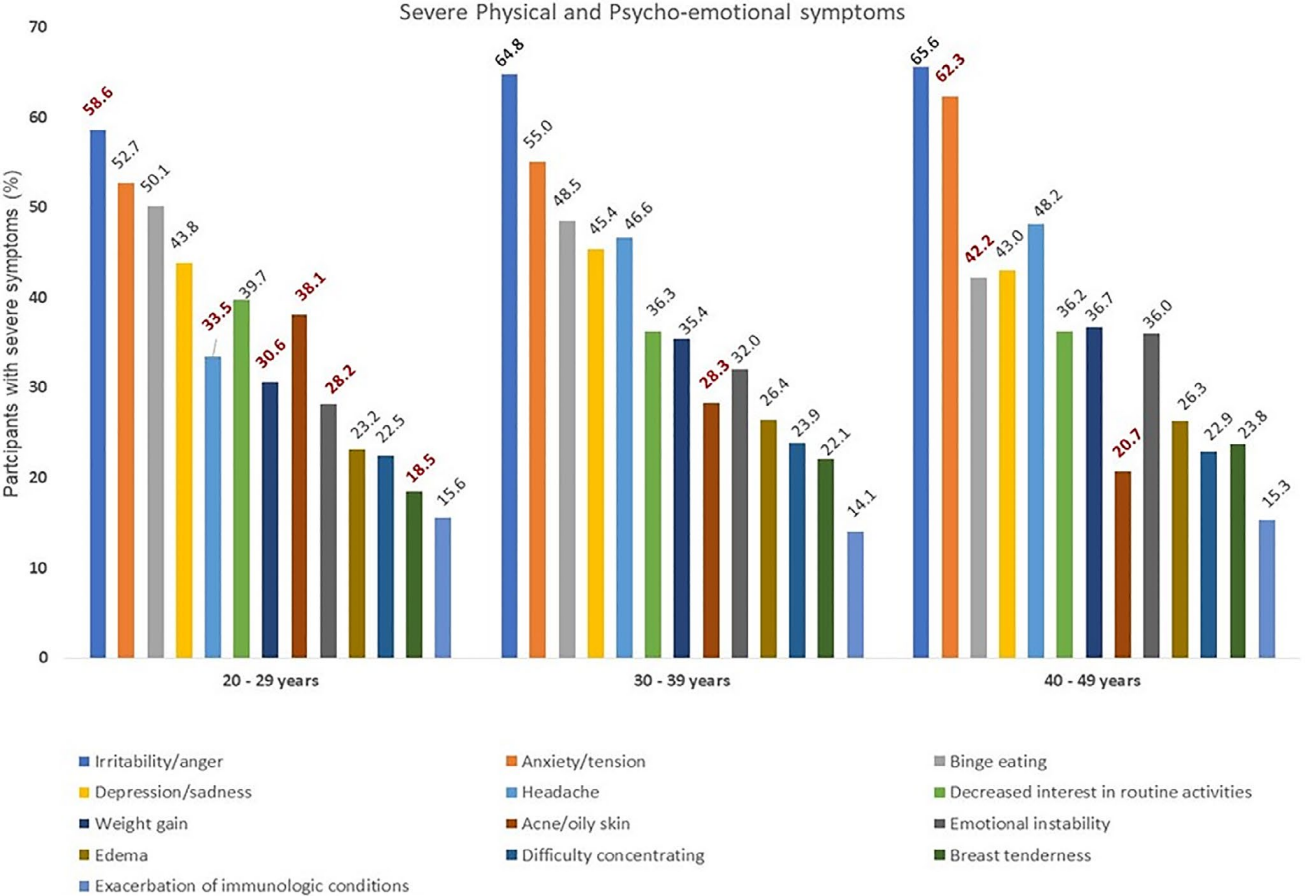
Comparison of severe physical and psycho‐emotional symptoms between age groups. Statistically significant differences are highlighted in bold and underlined.

Figure 2 summarizes these findings, illustrating the varying severity of symptoms among different age cohorts of women.

### 3.4 Willingness to use contraceptives for PMD treatment across age groups

**FIGURE 3:**
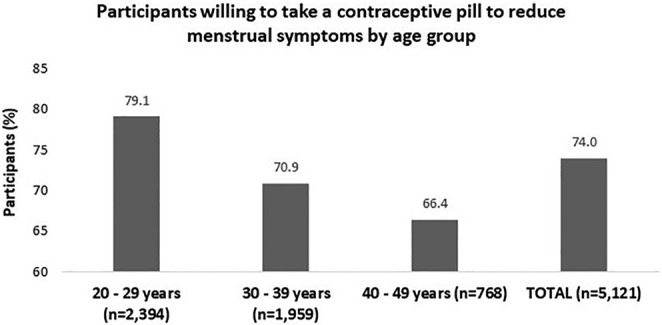
Willingness to use contraceptives for alleviating premenstrual symptoms by age group. *P* < 0.001.

On average, 3791 (74%) of participating women expressed willingness to use hormonal oral contraceptives for PMD symptom relief. Following adjustment for significance levels, the 20–29 year age group exhibited statistically significant differences compared to other age groups, demonstrating at least an 8% higher inclination to use contraceptives than the older age groups (*P* < 0.016) (Figure 3).

## 4 DISCUSSION

PMS is a common condition among reproductive‐age women and symptoms, both physical and psychological can affect women in all age groups, through menopause. In our nationwide study, premenstrual symptoms affected most of the women with a variation on symptoms intensity according to age group. Age significantly affects women's experiences of premenstrual symptoms.^9^ In adolescents, the likelihood of anovulatory cycles decreases with age, whereas it increases among premenopausal women. This variation in hormonal fluctuations prompts investigation into how perimenstrual symptoms vary with age.^13^

International studies have reported varying effects of age on psycho‐emotional and somatic symptoms associated with PMS.^8^, ^9^, ^14^, ^15^, ^16^, ^17^ A cross‐sectional study involving 7226 women from 14 countries revealed age‐related differences in symptom intensity, with younger and older women experiencing lower symptom intensity, with maximum intensity around age 35. Irritability was consistently among the top prevalent symptoms.^9^

In Brazil, a study of 1395 women aged 15–49 identified common PMS including irritability, abdominal discomfort, nervousness, headache, tiredness, and breast tenderness. Higher prevalence was noted among younger women (<30 years) and those not using hormonal contraception.^16^

Another observational study of 340 women (mean age 23.93 years ±9.41 years) reported dysmenorrhea in 63%, with fatigue affecting 32%, bloating affecting 18.9%, and back pain 13.3%. Irritability and anxiety were common, affecting 39.6% and 34.6%, respectively.^18^

Deuster et al. studied women aged 18–44 years and found severe symptoms such as extreme irritability (17.4%), backaches (14.2%), and bloating (13.2%). Symptom prevalence varied by age, with younger women more affected.^19^ Another study involving women aged 21–45 years demonstrated a decrease in symptom severity with increasing age, and symptom intensity was lower among users of oral contraceptives.^17^

Interview setting versus self‐administered questionnaires may influence reported findings. These studies lacked segregation of symptom prevalence by physical or psycho‐emotional domains and age groups. A Brazilian study also observed increasing severe PMD from 2.9% in 10–19‐year‐olds to 16.1% in 40–49‐year‐olds.^15^

A global survey of 238 114 app users aged 18–55 found age‐related differences in symptoms, with food cravings (85.28%), mood swings/anxiety (64.18%), and fatigue (57.3%) being the most reported. Symptoms such as absent‐mindedness, low libido and sleep changes increased with age (*P* < 0.001), whereas mood swings/anxiety levels were consistent across age groups. Approximately 28.61% of respondents reported that PMD symptoms, especially mood‐related issues, affected their daily lives during each menstrual cycle.^20^

The relationship between age and related symptoms remains unclear. Our study noted a significant correlation between older age and increased symptom severity. Logue and Moss observed that younger women experience more menstrual symptoms, while older women experience more premenstrual symptoms.^13^ Such differentiation may explain the variability across studies. However, our study did not explore this aspect specifically.

Older women (>40 years) are less inclined to use contraceptives for PMD treatment, despite 74% of participants expressing readiness to use contraceptives for managing premenopausal symptoms. Younger women are more willing, influenced by contraception needs and awareness of additional benefits. Combined oral contraceptives with drospirenone and ethinyl estradiol are more effective for PMD/PMDD due to their shorter hormone‐free interval (4 rather than 7 days) and spironolactone‐like activity of drospirenone. Extended or continuous regimens are beneficial for suppressing follicle development and maintaining stable hormone levels during PMD.^21^, ^22^, ^23^, ^24^, ^25^, ^26^, ^27^, ^28^

A systematic review of 1205 women aged 24.6 to 36.5 years with PMD or PMDD found that combined oral contraceptives improved overall premenstrual symptoms. However, there is insufficient evidence to determine which specific combined oral contraceptive is most effective for treating depressive symptoms associated to PMD.^29^

The main limitation of this study was its insufficient data on variables such as race/ethnicity, body mass index, food habits, and socioeconomic status. PMD has been associated with factors beyond age, including race, obesity, unhealthy eating habits, smoking, alcohol abuse, and mental stress.^16^, ^30^, ^31^, ^32^, ^33^, ^34^

Despite its retrospective, self‐reported approach and potential biases, the strength of the present study lies in its use of a validated, Brazil‐adapted questionnaire and a large nationwide sample, allowing for comprehensive assessment of PMD symptom prevalence and intensity across age groups and informing target PMD treatment strategies.

Understanding women's needs empower healthcare professionals and gynecologists enhance care by addressing PMD symptoms. This knowledge supports early diagnosis, effective treatment, and informed counseling on therapies such as contraceptives that reduce menstrual frequency, thus improving women's quality of life.

Moderate to severe PMD significantly impacts quality of life and imposes societal costs through reduced productivity, absenteeism, and increased healthcare utilization.^35^ Comprehensive studies across diverse populations are crucial for understanding PMD symptom prevalence, intensity and functional impairments, as well as aligning interventions with women's preferences. Despite its high prevalence, PMD is often overlooked by healthcare professionals and patients. Gynecologists frequently overlook these symptoms during evaluations, missing opportunities for effective treatments that could significantly improve quality of life. Furthermore, challenges persist in determining effective interventions due to resistance among patients and health providers towards antidepressants and hormonal contraceptives, despite their increasing utilization.^36^, ^37^

## 5 CONCLUSION

This study highlighted that psycho‐emotional symptoms such as irritability and anxiety were more pronounced than physical symptoms among Brazilian women of reproductive age experiencing PMS. Acne/oily skin was most prevalent in the 20–29 age group, while headaches were prevalent and severe among women aged 30–49 years. Younger women demonstrated a higher willingness to use contraceptives for PMS treatment, with oral contraceptives being the preferred choice for the majority (5121, 74%). These findings emphasize the importance of tailoring diagnostic and therapeutic approaches based on individual symptom profiles for women with PMS. Such personalized strategies have the potential to significantly improve women's quality of life, across personal, social and professional domains.

## AUTHOR CONTRIBUTIONS

Adriana O. Pedro: Contributed to design, data analysis and revision of the manuscript. Juliana D. P. Brandão: Contributed to data analysis and wrote the manuscript. Samantha B. O. Silva: Contributed to data analysis and wrote the manuscript. Maura G. Lapa: Contributed to the statistical and data analysis of the manuscript. Vivienne C. Castilho: Contributed to the design and revision of the manuscript.

## FUNDING INFORMATION

This study received financial support from Libbs Farmacêutica LTDA.

## CONFLICT OF INTEREST STATEMENT

Each author has confirmed compliance with the journal's requirements for authorship. Adriana O Pedro has served on the advisory board or has been a consultant for Libbs Farmacêutica, Abott, Achè, Amgen, EMS, Eurofarma, Grumenthal, Mantecorp‐Farmasa, and Sanofi. She has also served on the speaker's bureau for Libbs Farmacêutica, Abott, Achè, Amgen, EMS, Eurofarma, Grumenthal, Mantecorp‐Farmasa, and Sanofi‐Aventis. Juliana D. P. Brandao, Samantha B. O. Silva, and Maura G. Lapa and Vivienne C. Castilho are employed at Libbs Farmacêutica.

## Data Availability

Research data are not shared.
